# Racial/ethnic disparities among Asian Americans in inpatient acute myocardial infarction mortality in the United States

**DOI:** 10.1186/s12913-018-3180-0

**Published:** 2018-05-16

**Authors:** Eun Ji Kim, Nancy R. Kressin, Michael K. Paasche-Orlow, Lenny Lopez, Jennifer E. Rosen, Mengyun Lin, Amresh D. Hanchate

**Affiliations:** 1 General Internal Medicine, Zucker School of Medicine at Hofstra/Northwell, 2001 Marcus Avenue Suite S160, Lake Success, NY 11042 USA; 2 General Internal Medicine, Boston University School of Medicine, 801 Massachusetts Avenue, Crosstown Two, Boston, MA 02118 USA; 3 VA Boston Healthcare System, 150 S. Huntington Avenue, Boston, MA 02130 USA; 4 University of California San Francisco School of Medicine, 4150 Clement Street, San Francisco, CA 94121 USA; 5 MedStar Washington Hospital Center, 106 Irving Street NW POB South 124, Washington, DC 20010 USA

**Keywords:** Acute myocardial infarction, Mortality, comorbidities, Health disparity, Asian

## Abstract

**Background:**

Acute myocardial infarction (AMI) is a common high-risk disease with inpatient mortality of 5% nationally. But little is known about this outcome among Asian Americans (Asians), a fast growing racial/ethnic minority in the country. The objectives of the study are to obtain near-national estimates of differences in AMI inpatient mortality between minorities (including Asians) and non-Hispanic Whites and identify comorbidities and sociodemographic characteristics associated with these differences.

**Method:**

This is a retrospective analysis of 2010–2011 state inpatient discharge data from 10 states with the largest share of Asian population. We identified hospitalization with a primary diagnosis of AMI using the ICD-9 code and used self-reported race/ethnicity to identify White, Black, Hispanic, and Asian. We performed descriptive analysis of sociodemographic characteristics, medical comorbidities, type of AMI, and receipt of cardiac procedures. Next, we examined overall inpatient AMI mortality rate based on patients’ race/ethnicity. We also examined the types of AMI and a receipt of invasive cardiac procedures by race/ethnicity. Lastly, we used sequential multivariate logistic regression models to study inpatient mortality for each minority group compared to Whites, adjusting for covariates.

**Results:**

Over 70% of the national Asian population resides in the 10 states. There were 496,472 hospitalizations with a primary diagnosis of AMI; 75% of all cases were Whites, 10% were Blacks, 12% were Hispanics, and 3% were Asians. Asians had a higher prevalence of cardiac comorbidities, including hypertension, diabetes, and kidney failure compared to Whites (*p*-value< 0.01). There were 158,623 STEMI (ST-elevation AMI), and the proportion of hospitalizations for STEMI was the highest for Asians (35.2% for Asians, 32.7% for Whites, 25.3% for Blacks, and 32.1% for Hispanics). Asians had the highest rates of inpatient AMI mortality: 7.2% for Asians, 6.3% for Whites, 5.4% for Blacks, and 5.9% for Hispanics (ANOVA p-value < 0.01). In adjusted analyses, Asians (OR = 1.11 [95% CI: 1.04–1.19]) and Hispanics (OR = 1.14 [1.09–1.19]) had a higher likelihood of inpatient mortality compared to Whites.

**Conclusions:**

Asians had a higher risk-adjusted likelihood of inpatient AMI mortality compared to Whites. Further research is needed to identify the underlying reasons for this finding to improve AMI disparities for Asians.

## Background

Coronary artery disease is a common condition that affects 15 million adults in the US and about 715,000 people have acute myocardial infarction (AMI) annually [1]. AMI mortality has been significantly reduced with advances in care, standardization of AMI management, and modifications of AMI-related risk factors [2–4]. Despite these improvements, there exist significant racial and ethnic disparities in the treatment and outcome of AMI [5–21]. These studies have shown that minorities, mostly non-Hispanic Blacks and Hispanics, are less likely to have cardiac invasive procedures and have higher AMI mortality compared to non-Hispanic Whites. However, little is known about inpatient AMI mortality of Asians residing in the US due to limited numbers of national data that identify Asians (e.g. the National Health and Nutrition Examination Survey only started to identify Asians starting 2011).

Asian Americans (henceforth referred to as Asians), including Pacific Islanders, make up 4.8% of the US population and the population has been growing faster than the national rate [22]. Overall, Asians in the population have a relatively low prevalence of AMI-related comorbidities, including obesity, diabetes, smoking, and hypertension [23–26] and a relatively low national AMI mortality rate (age-adjusted AMI mortality rate from death certificate for non-Hispanic White men was 196.7/100,000 population and Asian men was 109.1/100,000 population in 1998) [27]. Despite studies that have examined health disparities involving Asians for other medical conditions such as stroke [28, 29], previous research looking at racial/ethnic disparities in AMI outcomes is limited. Many AMI studies have focused on non-Asian minorities in the US or involved Asians residing in Asian countries [29–32]. These studies showed mixed results, ranging from no differences to significant differences in outcomes.

To address the gaps in the literature on differences in AMI outcomes among Asian Americans and to provide updates in national AMI outcomes, we investigated the following research questions using comprehensive data from 10 states that together account for over 70% of the national Asian American (Asian) population: [1] Are there differences in observed AMI inpatient mortality between each of the minority groups (Asians, Blacks, or Hispanics) and Whites? [2] What is the risk-adjusted inpatient AMI mortality of minorities compared to Whites? [3] What are the sociodemographic and clinical characteristics associated with inpatient AMI mortality? By answering these questions, we want to understand racial/ethnic differences in inpatient AMI mortality as well as identify characteristics that are associated with differences in AMI inpatient mortality.

## Methods

### Study population

Our primary data was the 2010–2011 state inpatient discharge data, containing all patient discharges at non-federal short-term acute hospitals, from 10 US states - California, Florida, Illinois, Massachusetts, Maryland, New Jersey, New York, Pennsylvania, Texas, and Virginia. These states were selected based on having sizable Asian populations and the completeness of race/ethnicity reporting in the discharge data. Data for California, Texas, Illinois, Pennsylvania, Massachusetts, and Virginia were obtained from the respective state agencies [33], and data for remaining states were obtained from AHRQ HCUP Central Distributor [34]. The data was built and designed after the Feng et al. paper [35]. The selected 10 states contained 70% of the national Asian population (Appendix 1). We identified all discharges for adult patients aged 18 and older with the principal diagnosis of AMI (ICD-9: 410) [36]. We excluded 64,561 patients who were transferred to another short-term hospital, admitted with obstetric-related conditions, and missing key variables (discharge disposition, gender, age, discharge quarter/year, principal diagnosis, or admission information) [37].

### Race/ethnicity

We used self-reported race/ethnicity and categorized patients into the following race/ethnicity groups: Hispanics, (non-Hispanic) Whites, (non-Hispanic) Blacks, (non-Hispanic) Asians, Others or unknown (i.e., missing). For the subjects with missing race/ethnicity information (2.6% of the study population), race/ethnicity was re-assigned using the “hot-deck” statistical imputation method [38]. This method randomly assigns race/ethnicity for patients with missing race/ethnicity in the same proportion as the race/ethnicity distribution for those with known race/ethnicity. As previous studies have indicated that Others include a sizable proportion of Whites, we grouped Others with Whites in all the analyses [39].

### Covariates

Based on previous studies, we identified key demographics, comorbidities, types of AMI, and use of invasive cardiac procedures that were associated with AMI mortality. Socio-demographic variables included sex, age, and state of residence [17, 40–44]. We categorized age into seven groups: 18–34, 35–44, 45–54, 55–64, 65–74, 75–84, and 85+. We used Elixhauser categories to identify comorbidities and determined whether the patient had the comorbid condition or not (comorbid conditions include congestive heart failure, cardiac arrhythmias, valvular disease, pulmonary circulation disorders, peripheral vascular disease, hypertension, paralysis, other neurological disorders, chronic pulmonary disease, diabetes (uncomplicated), diabetes with chronic complications, hypothyroidism, renal failure, liver disease, peptic ulcer disease without bleeding, acquired immune deficiency syndrome, lymphoma, metastatic cancer, solid tumor without metastasis, rheumatoid arthritis/collagen vascular diseases, coagulopathy, obesity, weight loss, fluid and electrolyte disorders, blood loss anemia, deficiency anemias, alcohol abuse, drug abuse, psychoses, depression) [45]. Using secondary diagnosis codes reported in the index discharge records, the Elixhauser method identifies 30 risk groups associated with inpatient mortality; these are defined as 30 separate indicator (0/1) fields. For types of AMI, we differentiated AMI cases into non-ST-segment elevation (NSTEMI) (ICD-9 = 410.7) and ST-segment elevation MI (STEMI) (all ICD-9 = 410 excluding 410.7). We used diagnosis and procedure codes from the *International Classification of Diseases, Ninth Revision, Clinical modification* and corresponding *Current Procedural Terminology* to code for the invasive cardiac procedures of coronary artery bypass surgery (CABG) and percutaneous coronary intervention (PCI) [36].

### Primary outcome: Inpatient acute MI mortality

The main outcome was inpatient mortality from a hospitalization for AMI (ICD-9: 410). This is one of the Inpatient Quality Indicators measured by the Agency for Healthcare Research and Quality (AHRQ) [37].

### Statistical analysis

Statistical analysis was conducted using SAS software, version 9.3 (SAS Institute Inc., Cary, NC). We performed descriptive analysis of socio-demographic characteristics, medical comorbidities, and types of AMI based on patients’ race/ethnicity. We performed chi-square and t-tests to compare differences between each of the minority groups and Whites. Next, we examined use of PCI and CABG for overall AMI, and then among those with STEMI and NSTEMI.

We used multivariable logistic regression models to study differences in inpatient mortality among racial/ethnic minorities (Asians, Hispanics, and Blacks) compared to Whites. To better identify the source of differences in inpatient mortality, we estimated odds ratios in a sequence of models, adjusting for a wider array of covariates in the following order: age, gender, geographical location (state), co-morbidities (Elixhauser categories), type of AMI, and invasive cardiac procedures (CABG and PCI). We did not correct for multiple testing. Sensitivity analyses were performed to examine disparities in AMI mortality among those who were transferred from another short-term hospital, since this subgroup of patients may be systematically different in severity.

The Boston University Institutional Review Board approved this study.

## Results

We identified 561,041 AMI cases. Of these, we excluded 62,267 (11.1%) AMI admissions that were transferred to another hospital as we do not have information regarding their health outcomes (percentages of each racial/ethnic groups excluded: Asians = 14.5%, White = 10.9%, Black = 11.3%, and Hispanics = 11.3%). In addition, we excluded 2302 (0.4%) AMI cases with obstetric-related conditions or missing key variables. The study sample included 496,472 AMI cases.

### Socio-demographic characteristics

Table 1 shows the characteristics of the total sample as well as subgroups of the different racial/ethnic groups. The majority of the study population were White (75%), followed by 12% Hispanics, 10% Black, and 3% Asian. There was a significant difference in mean age and percentages of women in different racial/ethnic groups (ANOVA; *p*-value < 0.01 for both). Mean average age was the oldest for White (mean age = 69.4 years old) and the youngest for Black (mean age = 63.8 years old). Significantly higher percentage of Black patients (48.0%) and lower percentage of Asian patients (35.2%) were women compared to Whites (39.4%) (*p*-values< 0.05). There were wide variations in racial/ethnic composition in each of the 10 states.

**Table 1:** Socio-demographic characteristics of AMI hospitalizations by race/ethnicity^a^*

Variable	All (*n* = 496,472)	Asian (*n* = 14,977)	White (*n* = 372,556)	Black (*n* = 51,403)	Hispanic (*n* = 57,536)
Age					
Mean age, years old (SD)	68.3 (14.7)	67.2 (14.5)	69.4 (14.5)	63.8 (14.6)*	65.3 (14.4)*
Age groups (%)					
18–34	0.8	1.0	0.6	1.7	1.4
35–44	4.3	5.1	3.7	6.9	5.9
45–54	14.3	14.6	13.2	19.8	17.0
55–64	22.0	22.9	21.1	25.1	24.5
65–74	21.4	22.2	21.2	21.1	22.2
75–84	21.3	20.9	22.4	16.4	19.0
85+	15.9	13.3	17.9	9.0	10.0
Gender					
Women (%)	40.0	35.2*	39.4	48.0*	37.7
State of residence** (%)					
California	*n* = 92,285	9.6	63.6	7.2	19.6
Florida	*n* = 78,753	0.7	75.8	9.4	14.1
Illinois	*n* = 39,950	2.0	78.1	14.1	5.8
Massachusetts	*n* = 23,176	1.7	90.2	4.0	4.0
Maryland	*n* = 15,523	1.9	72.5	24.0	1.6
New Jersey	*n* = 29,662	3.0	76.8	11.3	8.8
New York	*n* = 59,521	2.7	78.9	10.7	7.8
Pennsylvania	n = 57,160	0.5	89.8	7.8	2.0
Texas	*n* = 74,553	1.3	66.3	10.9	21.6
Virginia	*n* = 25,889	1.6	67.6	18.3	1.5

^a^Values are means (standard deviation) or percentages of patients

*P-value< 0.05 when each of the racial/ethnic group (Asian, White, or Black) was compared to White

**Racial/ethnic distribution in each state

### Clinical characteristics

We examined the prevalence of each of the comorbidities in the Elixhauser comorbidity index (Table 2). Compared to Whites, Asians had a mixed profile of comorbid conditions, with lower prevalence of some conditions (obesity, cardiac arrhythmia, and peripheral vascular disease), but higher prevalence of a wider range of conditions (congestive heart failure, diabetes, hypertension, renal failure, and coagulopathy) (all *p*-values were less than 0.01). There were significant racial/ethnic differences in the average length of stay and number of Elixhauser comorbidity diagnoses (ANOVA; both *p*-value < 0.01). Among different groups, each of the minority groups had longer average length of stay and Blacks had the highest average number of diagnoses (11.6 diagnoses) compared to Whites (Table 3).

**Table 2:** Prevalence of co-morbidities (Elixhauser comorbidities) by race/ethnicity

Variable	Asian (*n* = 14,977)	White (*n* = 372,556)	Black (*n* = 51,403)	Hispanic (*n* = 57,536)
% of AMI hospitalizations with the comorbidities				
Congestive heart failure	34.5	31.9	35.5	32.0*
Cardiac arrhythmias	36.5	39.5	31.8	31.3
Peripheral vascular disease	9.4	11.3	11.2*	10.6
Hypertension	75.7	70.5	82.4	75.9
Chronic pulmonary disease	13.6	20.5	18.5	14.9
Diabetes, uncomplicated	33.6	26.4	34.1	38.8
Diabetes with chronic complications	11.7	5.7	9.0	10.3
Renal failure	26.6	18.3	28.1	22.0
Coagulopathy	7.4	5.0	4.9*	5.2*
Deficiency anemias	23.1	15.4	21.8	19.4

*P-value ≥ 0.05 when each of the racial/ethnic group (Asian, White, or Black) was compared to White (unmarked signify *p*-value < 0.05)

**Table 3:** Clinical characteristics of AMI hospitalizations by race/ethnicity^a^

Variable	Asian	White	Black	Hispanic
Number of diagnoses (SD)	11.3 (6.2)	11.3 (5.2)	11.6 (5.2)*	10.8 (5.2)*
Length of stay, days (SD)	6.2 (7.4)*	5.3 (17.4)	5.6 (7.0)*	5.9 (19.5)*
Types of AMI				
STEMI	35.2*	32.7	25.3*	32.1*
NSTEMI	64.8*	67.3	74.7*	67.9*
Receipt of invasive cardiac procedures				
PCI	48.0	48.2	41.0*	47.6*
CABG	10.6*	9.3	6.5*	10.2*
Receipt of invasive cardiac procedures by AMI type				
STEMI (*n* = 158,623)				
PCI	69.5	72.1	68.1*	72.2
CABG	8.0*	7.3	5.4*	8.1*
NSTEMI (*n* = 337,849)				
PCI	36.3	36.5	31.9*	35.9*
CABG	12.0*	10.2	6.9*	11.3*

^a^Values are means (standard deviation) or percentages of patients

*P-value< 0.05 when each of the racial/ethnic group (Asian, White, or Black) was compared to White

Next, we differentiated type of AMI; there were 337,849 (68.0%) hospitalizations for NSTEMI and 158,623 (32.0%) hospitalizations for STEMI (Table 3). Significantly higher proportion of Asians (35.2%) had STEMI and lower proportions of Blacks (25.3%) or Hispanics (32.1%) had STEMI compared to Whites (32.7%) (*p*-values < 0.05). We also examined the prevalence of cardiac procedure use, specifically percutaneous coronary intervention (PCI) and coronary artery bypass graft (CABG). Among AMI hospitalization by Asians, 48.0% received PCI and 10.6% received CABG; this is similar for PCI and higher for CABG compared to Whites. Regardless of type of procedure or AMI, Blacks had the lowest rate of cardiac procedures during the hospitalization. In regards to mortality after cardiac procedures (PCI or CABG), Asians had higher mortality compared to non-Asians (*p* < 0.01), specifically after PCI (Appendix 2).

### Observed inpatient AMI mortality

There were significant differences in observed inpatient AMI mortality among different racial and ethnic groups. Asians had the highest observed inpatient AMI mortality (inpatient mortality Asian = 7.2%; White = 6.3%; Black = 5.4%, and Hispanic = 5.9%). This finding persisted for both STEMI (inpatient mortality Asian = 10.4%; White = 9.4%; Black = 9.8%, and Hispanic = 8.8%) and NSTEMI (inpatient mortality Asian = 5.4%; White = 4.8%; Black = 3.9%, and Hispanic = 4.6%).

### Models of inpatient AMI mortality adjusting for covariates

We examined the racial/ethnic differences in AMI mortality using a sequence of multivariable logistic models, cumulatively including covariates from the different domains (Table 4). Model 1 (which only included race/ethnicity variable) reported higher unadjusted AMI mortality among Asians (odds ratio (OR) = 1.16 [95% CI: 1.09–1.23]) compared to Whites; in contrast, Blacks (OR = 0.85 [0.81–0.88]) and Hispanics (OR = 0.94 [0.91–0.97]) had lower mortality. After adjusting for age differences (model 2), all minority groups (Asian OR = 1.27 [95% CI: 1.19–1.35], Black OR = 1.06 [95% CI: 1.02–1.11], and Hispanic OR = 1.11 [95% CI: 1.07–1.15]) had higher mortality compared to Whites. This trend persisted even after adjusting for gender, state of residence, and comorbidities; however, the Asian versus White gap decreased after accounting for differences in comorbidities (Model 5). When type of AMI was included in the analysis (Model 6), likelihood of AMI mortality increased and became significant for Blacks compared to Whites. In the final model (Model 7), there was a shift in AMI mortality among Blacks (from OR = 1.12 [1.07–1.17] to OR = 1.02 [95% CI: 0.98–1.06]) when invasive cardiac procedures were included in the model. Including the cardiac procedure covariate did not change the significance of AMI mortality of either Asians (OR = 1.11 [1.04–1.19]) or Hispanics (OR = 1.14 [1.09–1.19]) when compared to Whites. Sensitivity analysis looking at the subgroup of AMI hospitalizations of patients transferred from another short-term hospital (*n* = 81,888) showed no significant difference in inpatient AMI mortality between minorities and White (Asian OR = 0.86 [0.68–1.08]; Black OR = 0.94 [0.82–1.08]; and Hispanic OR = 1.02 [0.89–1.16]) after adjusting for covariates.

**Table 4:** Odds ratio [95% confidence interval] of inpatient AMI mortality associated with race/ethnicity (reference: non-Hispanic White)

	Asian	Black	Hispanic
	(*n* = 14,977)	(*n* = 51,403)	(*n* = 57,536)
Model 1: Unadjusted	1.16 [1.09–1.23]	0.85 [0.81–0.88]	0.94 [0.91–0.97]
Model 2: Model 1+ Age group	1.27 [1.19–1.35]	1.06 [1.02–1.11]	1.11 [1.07–1.15]
Model 3: Model 2+ Gender	1.27 [1.19–1.35]	1.06 [1.02–1.11]	1.11 [1.07–1.15]
Model 4: Model 3+ Geography (state)	1.21 [1.14–1.30]	1.05 [1.00–1.09]	1.09 [1.05–1.13]
Model 5: Model 4+ Patient comorbidities (Elixhauser grouping)	1.13 [1.06–1.21]	1.04 [1.00–1.09]	1.15 [1.10–1.19]
Model 6: Model 5+ Type of AMI	1.12 [1.04–1.20]	1.12 [1.07–1.17]	1.17 [1.12–1.22]
Model 7: Model 6+ Receipt of PTCA or CABG	1.11 [1.04–1.19]	1.02 [0.98–1.06]	1.14 [1.09–1.19]

## Discussion

Using comprehensive AMI discharge data from 10 states that contains 70% of the U.S. population of Asians, we found that Asians had the highest observed inpatient AMI mortality. There were differences in sociodemographic and clinical characteristics among different racial/ethnic groups. In risk-adjusted inpatient mortality, the extent of the mortality differences was reduced after adjusting for comorbidities, an indication of higher prevalence of comorbidities among Asians admitted with AMI. In the final model adjusting for sociodemographic characteristics, comorbidities, type of AMI, and invasive cardiac procedure use, Asians and Hispanics remained to have increased likelihoods of inpatient mortality compared to Whites.

One plausible explanation for the higher AMI inpatient mortality among Asians is higher disease burden and severity, specifically among those with elevated risk of AMI. As noted, there was a sizable reduction in the AMI mortality among Asians after adjusting for observed comorbidities, which indicates higher overall comorbidity burden. This pattern of higher comorbidity risk among Asian AMI hospitalizations contrasts with epidemiologic evidence of lower disease burden among the general population of Asians [23–26]. The population level death rate from AMI is lower among Asians, compared to Whites [27]; this is not inconsistent with our finding of higher mortality among hospitalizations for AMI, since our study does not take into account the risk of being hospitalized (for AMI). The higher cardiovascular risk among some Asians may partly be explained by their recent adoption of western diet and habits in the U.S. as well as worldwide [46, 47]. The shift in cardiovascular risk factors are associated with acculturation, particularly the number of years residing in the US [48–50]. Given no significant differences in likelihoods of AMI mortality among those who were transferred from another short-term hospital, disparities are not due to racial/ethnic differences in the proportion of patients who were transferred across hospitals. Instead, disparities were concentrated largely among cases where patients were treated in the hospitals where they were initially admitted.

Besides comorbidities, we observed higher AMI inpatient mortality among Asians who had cardiac procedures. We do not have information regarding decisions behind why patients received cardiac procedures but the high cardiac procedure rate among Asians might be due to more advanced or severe cases that require invasive interventions. The higher mortality among those receiving cardiac procedures needs further examination, as identifying contributing factors can improve future outcomes. If the high mortality is due to a high comorbidity burden, future studies should focus on re-stratifying risk for all AMI patients. However, if the high mortality is coming from procedure-related complications common among Asians, such as high bleeding risk after anti-platelet therapy [51–53], then different medical therapies, such as lower doses of antithrombotic medications, should be used. The absence of data about complications is a limitation of our study, thus we could not examine this further. Also, Asians with AMI may seek medical care when cardiac symptoms are severe and have been present for longer durations. Delays in receiving care have been associated with poor outcomes [54–56]. In a study that examined AMI patients experiencing symptoms for less than 24 h, there was no difference in mortality between Asians and Whites [32]. Our study did not differentiate patients based on their duration of symptoms, and the high mortality could have originated from higher inpatient mortality among Asians with longer duration of symptoms.

Another explanation is that certain Asian subgroups are contributing more to the high inpatient AMI mortality. Asians encompass diverse population and studies have identified heterogeneity in cardiovascular disease risk factors among Asians by country of origin. For example, previous studies have identified that South Asians have more cardiovascular risk factors and worse outcomes compared to Whites [57–59]. In addition, another study found that certain Asian and Hispanic ethnic groups have significantly lower insurance rates [60] and it is possible that these small groups of Asians with disadvantageous socioeconomic factors may experience a significantly greater impact on AMI mortality due to limited healthcare access and decreased preventive care visits. Lastly, the presence of language barriers among Asians with limited English proficiency could contribute to poor health outcomes [61, 62], and possibly higher AMI mortality.

There are several limitations of this retrospective observational study. Given our observational data, a causal relationship cannot be established between being Asian and increased inpatient AMI mortality. Due to the nature of secondary, administrative data, information on clinical patient status is limited. For example, clinical information regarding duration or severity of symptoms, admission vitals, EKG findings, and procedure complications could have provided further insight. The data also did not include medications, which would have been helpful in understanding race/ethnicity-specific medical management of AMI, or how clinical decisions were made in obtaining invasive cardiac procedures. Previous research has shown mixed results as to whether racial differences existed in decision-making involving invasive cardiac procedures [14, 63]. We are missing data in some states on other covariates such as health insurance status, educational achievement, and household income, which could affect patients’ access and decision to seek medical care for AMI. Our data also does not contain acculturation information, which could indirectly provide immigrants’ newly adopted cardiovascular risks. The findings may not be generalizable to states with small Asian populations, since we only included 10 states with a sizeable Asian population. Lastly, there has been a significant increase in multiracial populations, especially Asians tied to other races or ethnicities [22]. For our study, we used self-reported race/ethnicity, which did not identify multiracial patients.

## Conclusion

These data are among the first to show that Asian patients hospitalized with acute myocardial infarction had higher observed AMI mortality than Black, Hispanic, or White patients. The findings may have been driven by higher disease severity, heterogeneous risk-associated with Asian subgroups, and delayed medical care. We observed higher inpatient AMI mortality among Asians compared to Whites even after adjusting for sociodemographic and clinical characteristics. With the increasing number of Asians in the US, disparities in AMI mortality will become more significant. Further research should focus on patient-level details to better understand the heterogeneity of the Asian population and clinical factors associated with such disparities.

## Appendix 1

**Table 5:** 2010–2011 Census population and crude state-level counts of AMI by race/ethnicity (Age 18+)

	Crude state-level counts of AMI					State share (%) of national census population of adults, 2010				
State	Asian	White	Black	Hispanics	All	Asian	White	Black	Hispanics	All
California	8815	58,703	6673	18,094	92,285	33.1%	7.9%	6.1%	27.8%	11.9%
New York	1610	46,953	6338	4620	59,521	9.7%	5.8%	8.2%	7.3%	6.4%
Texas	949	49,415	8105	16,084	74,553	6.6%	5.8%	7.6%	18.4%	7.8%
New Jersey	899	22,792	3359	2612	29,662	4.9%	2.6%	3.1%	3.3%	2.9%
Illinois	784	31,217	5648	2301	39,950	4.0%	4.1%	4.8%	3.9%	4.1%
Florida	522	59,677	7423	11,131	78,753	3.1%	5.8%	7.6%	9.4%	6.3%
Virginia	425	20,340	4744	380	25,889	3.0%	2.6%	4.1%	1.3%	2.6%
Massachusetts	398	20,912	936	930	23,176	2.4%	2.6%	1.1%	1.2%	2.2%
Maryland	295	11,248	3731	249	15,523	2.2%	1.6%	4.5%	1.0%	1.9%
Pennsylvania	280	51,299	4446	1135	57,160	2.4%	5.2%	3.5%	1.4%	4.2%
Total	14,977	372,556	51,403	57,536	496,472	76.0%	50.4%	52.6%	84.6%	56.8%

## Appendix 2

**Table 6:** Percentage of patients receiving invasive cardiac procedure treatment for AMI and their observed inpatient mortality

Variable	Asian	White	Black	Hispanic	*P*-value*
AMI (*n* = 496,472)					
PCI or CABG	57.6%	56.5%	47.0%	56.8%	< 0.01
Mortality	3.7%	3.1%	2.9%	3.4%	< 0.01
PCI	48.0%	48.2%	41.0%	47.6%	0.09
Mortality	3.6%	3.0%	2.7%	3.4%	0.01
CABG	10.6%	9.3%	6.5%	10.2%	< 0.01
Mortality	4.6%	3.9%	4.6%	3.4%	0.15

*Comparing Asians versus non-Asians (combined non-Hispanic White, non-Hispanic Black, and Hispanic)

## Abbreviations

AHRQ: Agency for Healthcare Research and Quality
AMI: Acute myocardial infarction
ANOVA: Analysis of variance
Asian: Asian American
CABG: Coronary artery bypass graft
CPT: Current procedural terminology
ICD: International Classification of Disease
NSTEMI: Non-ST-segment elevation myocardial infarction
OR: Odds ratio
PCI: Percutaneous coronary intervention
STEMI: ST-segment elevation myocardial infarction
